# The Role of Visual Information in Body Size Estimation

**DOI:** 10.1177/2041669518796853

**Published:** 2018-09-05

**Authors:** Anne Thaler, Michael N. Geuss, Betty J. Mohler

**Affiliations:** Max Planck Institute for Biological Cybernetics, Tübingen, Germany

**Keywords:** visual perspective, body size estimation, body image, mirror, body perception

## Abstract

The conscious representation of our physical appearance is important for many aspects of everyday life. Here, we asked whether different visual experiences of our bodies influence body width estimates. In Experiment 1, width estimates of three body parts (foot, hips, and shoulders) without any visual access were compared to estimates with visual feedback available in a mirror or from a first-person perspective. In the no visual access and mirror condition, participants additionally estimated their head width. There was no influence of viewing condition on body part width estimates. Consistent with previous research, all body part widths were overestimated with greater overestimation of hip and head width. In Experiment 2, participants estimated the size of unfamiliar noncorporeal objects to test whether this overestimation was partially due to the metric body size estimation method or our experimental conditions. Object width was overestimated with visual feedback in a mirror available as compared to when directly looking at the object, but only for objects placed at shoulder and head height. We conclude that at least some of the overestimation of body part width seems to be body specific and occurs regardless of the visual information provided about the own body.

## Introduction

How our body is mentally represented in terms of visual properties can have a great influence on our psychological health. The conscious representation of our physical appearance is often referred to as our body image. Body image is characterized as a unity of present body sensations linked with social and perceptual experiences in the past and allows comparing one’s physical appearance to others. It is traditionally distinguished from body schema, an unconscious representation of the bodily dimensions used for action control (Dijkerman & De Haan, 2007; Paillard, 1999). Generally, body image is described as consisting of two components that are largely independent of one another, an attitudinal and a perceptual component (Gardner & Brown, 2014; Slade, 1994). The attitudinal component comprises feelings and thoughts that are placed on the whole body or body parts as a result of, for example, sociocultural norms of body ideals (Grabe, Ward, & Hyde, 2008), social comparison (Myers & Crowther, 2009), and appearance-related feedback from others. Negative social feedback and excessive appearance-related social comparison have been linked to poor psychological health and the development of body image and eating disturbances (J. K. Thompson, Coovert, & Stormer, 1999). Most research on the perceptual component of body image has used visual body size estimation tasks to investigate how veridically people estimate their own bodily dimensions. It is not clear yet whether and how nonvisual bodily information influences visual estimates of own body size. However, there are multiple factors suggesting that one’s body image is largely influenced by the visual experience with one’s own body, such as the higher spatial resolution of the visual system as compared with the somatosensory system, and the fact that perception of others’ bodies and thus self-other comparisons appear to be guided by vision.

It is currently unknown how different visual experiences that one has in daily life contribute to the mental image of one’s own body. Which visual information is predominantly used to form people’s perceptual body image? One’s body image could be formed by information about the bodily dimensions from the visual experiences with our body from a first-person perspective—as we see our body most of the time—or the third-person perspective through indirect media such as mirrors, reflections, or pictures.

The goal of this research was to investigate how two different visual perspectives on one’s body (first- and third-person) contribute to the mental representation of own bodily dimensions that is assessed in a body size estimation task. Understanding how different visual experiences may, or may not, influence the visual representation of own body size could improve our theoretical understanding of how body representations are formed and thus provide a fruitful and novel area to understanding differences between clinical and nonclinical populations with body image disturbances. To motivate this work, we briefly review empirical research using body size estimation tasks, the sparse literature on how different experiences contribute to body size perception, and some indications that body size estimations may be distorted when viewing one’s own body in a mirror.

### Empirical Research on Body Size Estimation

Several methods have been developed aiming at externalizing one’s body image to measure its dimensions. As the traditional concept of body image is that of a visual body representation, the methods rely on visually comparing dimensions of one’s own body to a reference. Two types of body size estimation methods are distinguished: metric and depictive body size estimation methods (Longo & Haggard, 2012). In metric methods, often also referred to as body part methods, the size of body parts is estimated against a spatial measure (e.g., a caliper or a measure tape) and thus local spatial estimates of body parts are assessed (Home, Van Vactor, & Emerson, 1991; Slade & Russell, 1973). In depictive methods, often also referred to as whole body methods, the size and shape of the whole body are compared with another body of self or other identity (e.g., photographs or computer-generated bodies) usually manipulated in body weight (Mölbert et al., 2018; Piryankova et al., 2014; Thaler et al., 2018; Tovée, Benson, Emery, Mason, & Cohen-Tovée, 2003). A recent review suggests that healthy people on average accurately estimate their own bodily dimensions in depictive methods, whereas they overestimate in metric methods (Mölbert et al., 2017).

Although it is often argued that body size estimation tasks assess the perceptual component of body image, the estimates were found to be influenced by cognitive-affective factors. Specifically, findings show that the accuracy of body size estimation correlates with attitudes toward the body in healthy women (Ben-Tovim, Walker, Murray, & Chin, 1990), and the degree of overestimation of certain body parts is influenced by body dissatisfaction and self-esteem in anorexia nervosa (Garner & Garfinkel, 1982). In line with this, different task instructions were found to have an effect on the estimates of own body size (Bowden, Touyz, Rodriguez, Hensley, & Beumont, 1989).

Overall, this research suggests that the size of own body parts is often overestimated in metric body size estimation methods and the degree of overestimation might be influenced by affective factors. However, it does not address how the mental image of our body is formed. What visual experience do we have with our own body that may allow us to make body-related self-other comparisons? We hypothesize that social comparison is best served by representing our body in a manner that is consistent with judging and comparing to others’ bodies, namely, from a third-person perspective. Specifically, viewing ourselves through, for example, a mirror allows a perspective on our own body that may be the basis of the mental image of our body.

### Body Size Estimation With Mirrors

A particularly salient visual experience of one’s body is viewing it through a mirror. Mirrors have been used to investigate body size estimation, especially in individuals with eating disorders and obesity, and are often used as a therapy tool to improve self-body evaluation through mirror confrontation (Cappon & Banks, 1968; Delinsky & Wilson, 2006; Garfinkel, Moldofsky, Garner, Stancer, & Coscina, 1978; Norris, 1984).

Some researchers have argued that body size estimation tasks assess memory of own body size rather than perception (Farrell, Shafran, & Fairburn, 2003; Shafran & Fairburn, 2002; Smeets, 1997). To address this issue, some studies have investigated body size estimation with visual feedback in a mirror available (Ben-Tovim & Walker, 1990; Gardner, Gallegos, Martinez, & Espinoza, 1989). For example, Ben-Tovim and Walker (1990) asked healthy adolescent girls to estimate the width of their bodies at several heights (face, shoulder, chest, waist, and hip) by adjusting the distance between two moveable light sources with and without mirror feedback. In the mirror condition, participants could see their mirror image in a full-length mirror that was placed adjacent to the moveable caliper apparatus while making the estimates. The results showed that participants significantly overestimated all body widths when compared to measurements of their actual body in both the mirror and the no mirror condition. The mirror feedback only influenced width estimates of the shoulders, such that overestimation was smaller than without mirror feedback. Similarly, Gardner et al. (1989) had obese and normal weight participants match the body size of a previously taken photograph shown on a monitor in the presence and absence of a mirror. There was no difference in results between the two groups; however, overall participants were more accurate in estimating their body size when the mirror was present.

In other studies, participants were presented with an image of themselves that was projected next to a full-length mirror with the projection having the same height as the mirror reflection (half of the physical body size). Participants’ task was to adjust the projected image such that it corresponded to their mirror reflection (Farrell et al., 2003; Shafran & Fairburn, 2002). In addition, Farrell et al. (2003) compared body size estimates to a condition in which participants had to adjust the size of an image projection of their body in life size to match their own body size from memory. Contrary to expectations, the results showed that participants overestimated their body size significantly more in the mirror condition compared to the memory condition. The authors argued that this might have been due to the different sizes of stimuli that were used in the two conditions. Using the same method but matching the image size between both conditions, Øverås, Kapstad, Brunborg, Landrø, and Lask (2014) found that patients with anorexia nervosa overestimated their body size more than controls in both the memory and perception condition. As in Farrell et al. (2003), they found that body size was overestimated more in the mirror compared with the memory condition but only for patients and not for healthy controls. Docteur, Urdapilleta, and Duarte (2012), on the other hand, found that healthy women accurately estimated their body size using a similar mirror-based method, but they overestimated in the memory condition. Furthermore, when adjusting an image so it matched a printed photograph of themselves, participants made accurate estimates.

Overall, these studies present inconclusive results as to whether perception and memory of own body size differ and how exactly they are distorted. Furthermore, most previous studies using a depictive body size estimation method do not allow to draw conclusions about differences in size estimation of single body parts because the body could only be adjusted as a whole, namely, in body weight, and not independently for single body parts (see, however, Gardner, Martinez, Espinoza, & Gallegos, 1988; McCabe, Ricciardelli, Sitaram, & Mikhail, 2006; Tovée et al., 2003). So far, none of this work has related estimates of body size in a mirror to those from memory and from a first-person perspective—as we see our body most of the time.

### Overview of the Experiments

The goal of the current experiments was to investigate how different visual experiences may contribute to the conscious representation of our bodily dimensions. In Experiment 1, we compared estimates of body widths without any visual access with estimates made when viewing one’s body in a mirror or from a first-person perspective. If estimates of own body size without visual access are more similar to those made when seeing one’s body in a mirror as compared to the first-person perspective, then it would suggest that participants’ mental representation of their bodily dimensions might partially be influenced by visually experiencing one’s body from a third-person perspective. In Experiment 2, we compared estimates of object widths placed at the height of the same body parts as in Experiment 1, when viewing the objects in a mirror and when directly looking at the objects.

## Experiment 1

### Methods

#### Participants

Forty-eight (32 female, 16 male) people from the Tübingen community participated in the experiment. All participants were naive to the purpose of the experiment and had normal or corrected-to-normal vision. The experiment was approved by the ethics committee of the University of Tübingen and was performed in accordance with the Declaration of Helsinki. Participants gave written informed consent and were compensated with €8 per hour for their participation.

#### Apparatus

In the Mirror condition, participants viewed their reflection in a 34 cm × 110 cm (width × height) sized standard mirror, mounted on a wall with a floor offset of 70 cm, 1.75 m from the viewers’ location. The mirror was tested, and no obvious distortions were found. For the visual matching task, a standard 3 m steel tape measure was used with metric values displayed only on one side (experimenter), the opposite side (participant) was blank.

#### Design

**Figure 1.**
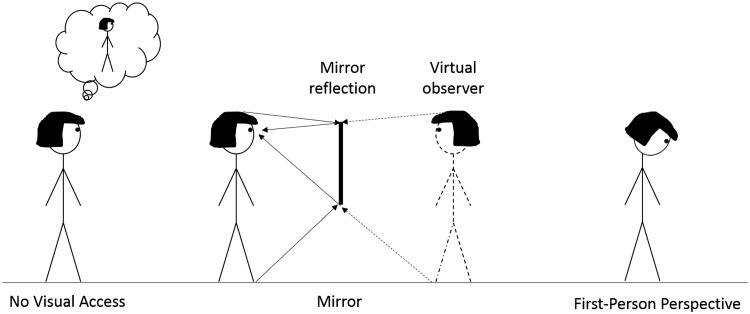
Overview of the three experimental conditions in Experiment 1. Participants either estimated their body size without visual access with visual feedback in the mirror available or from a first-person perspective.

A mixed design was implemented. Condition (Mirror, No Visual Access, and First-Person Perspective; Figure 1) was manipulated between participants. All participants estimated the width of three body parts (foot, hips, and shoulders) and participants in the Mirror and No Visual Access conditions additionally estimated the width of their head. Participants in the First-Person Perspective condition did not estimate the width of their head as it is impossible to view this dimension when looking down at one’s body. The width of every body part (foot, hip, shoulder, and head) was estimated twice, once with the tape measure starting small and once starting long. Participants were instructed to estimate the width of their right foot at the widest point, of their hips at the widest point, of their shoulders at the point at which they begin to curve down, and of their head at the cheek bones. In the Mirror condition, participants estimated the physical width of each body part as seen in the mirror but were explicitly instructed that they were not allowed to look down or touch their body parts with their hands. In the No Visual Access condition, participants estimated the physical width of each body part as they remembered it but were not allowed to look down at their body, touch specific body parts, or look into a mirror. In the First-Person Perspective condition, participants estimated the physical width of each body part from looking down but were not allowed to touch specific body parts or look into a mirror. The experimenter verified that the participants did not violate the instructions. The order of body part estimates was completely randomized without restriction.

#### Procedure

After filling out the informed consent form, participants were randomly assigned to one of the three conditions. To estimate the size of each body part, participants performed a metric body size estimation task (visual matching task) whereby the experimenter would either slowly extend or contract the length of a tape measure. The numbers of the tape measure were not visible to the participant. The participants’ task was to adjust the length of the tape measure through verbal instructions so that it corresponded to the width of the body part currently being estimated. They were free to adjust the length of the tape measure as often as necessary. After participants had completed the experiment, they were asked about their thoughts about the hypothesis of the experiment and the widths of all estimated body parts were measured. The experiment lasted approximately 30 min.

### Analysis

For getting a measure of the accuracy of the body part width estimates, a body perception index (BPI) was calculated according to the formula: BPI = (estimated size/actual size) × 100 (Slade & Russell, 1973). To analyze the influence of viewing condition (No Visual Access, Mirror, and First-Person Perspective) on the BPI of the different body parts, a multilevel regression analysis was conducted. This analysis was chosen over an analysis of variance as it can effectively deal with missing data points and therefore allows to analyze the BPI for all body parts and conditions simultaneously even though participants did not estimate their head width in the First-Person Perspective condition. The analysis was done using the lmer function of the lme4 package in R (Bates, Mächler, Bolker, & Walker, 2014). The BPI was regressed onto the body part (foot, hip, shoulder, and head), and the condition (No Visual Access, Mirror, and First-Person Perspective). All factors were allowed to interact. Specifically, the following mixed-effects model was fitted (in Wilkinson notation; Wilkinson & Rogers, 1973): BPI ∼ body part + condition + body part: condition + (1|participant). The results reported are with Satterthwaite approximation for degrees of freedom. For the pairwise comparisons, *t* tests with Bonferroni corrections were used because in the mixed-effects model the predicted value at combinations with the head could not be estimated due to the head width not being estimated in the First-Person Perspective condition. Effect sizes are reported as Cohen’s *d*.

### Results

**Figure 2.**
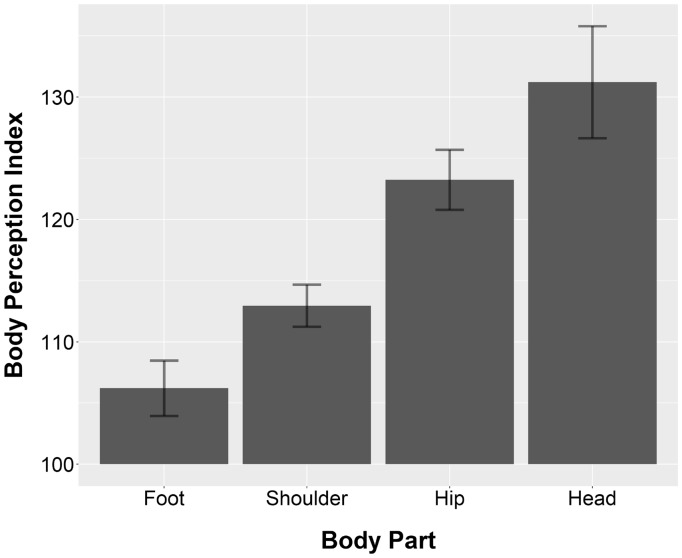
Body perception index as a function of body part, collapsed across the three experimental conditions of Experiment 1. Values higher than 100 indicate overestimation in terms of percentage of the actual body size. Error bars represent one standard error from the mean.

The multilevel analysis revealed that there was no main effect of condition (No Visual Access, Mirror, and First-Person Perspective), *F*(2, 50.26) = 1.32, *p* = .28, and also no interaction between condition and body part, *F*(5, 124.13) = 1.68, *p* = .14, suggesting that the accuracy of the body width estimates did not differ across conditions. However, there was a main effect of body part, *F*(3, 124.38) = 18.79, *p* < .001. Pairwise comparisons using independent *t* tests with Bonferroni *p* value corrections showed that the BPI for the foot (*M* = 106.21, standard deviation [*SD*] = 15.99) and shoulder (*M* = 112.94, *SD* = 12.16) was smaller than the BPI for the hip (*M* = 123.23, *SD* = 17.35) and head (*M* = 131.21, *SD* = 25.05); foot–hip: *p* < .001, *d* = 1.02; foot–head: *p* < .001, *d* = 1.26; shoulder–hip: *p* = .02, *d* = 0.69; shoulder–head: *p* < .001, *d* = 1.01. There was no difference in BPI between the foot and shoulder (*p* = .32, *d* = 0.47) and between the hip and head (*p* = .28, *d* = 0.39). The results are shown in Figure 2. To examine whether the BPI for the different body parts significantly differed from accurate size estimation (BPI = 100), one-sample *t* tests were conducted. The results showed that the size of all body parts was significantly overestimated: foot: *t*(49) = 2.74, *p* = .008, *d* = 0.39; shoulder: *t*(49) = 7.53, *p* < .001, *d* = 1.06; hip: *t*(49) = 9.47, *p* < .001, *d* = 1.34; and head: *t*(29) = 6.82, *p* < .001, *d* = 1.25.^1^

### Discussion

The results of Experiment 1 show no effect of viewing condition on the accuracy of body width estimates. We found, however, that the width of all body parts was significantly overestimated. This finding is in line with a recently published review on body size estimation suggesting that healthy people tend to overestimate their body dimensions in metric body size estimation tasks similar to the one used here (Mölbert et al., 2017). Mölbert et al. (2017) suggest that metric tasks might also recruit more implicit body representations, for example, from proprioception, that are used for action control. Part of this overestimation of body width could be that when navigating through space, we often leave a margin of error (Warren & Whang, 1987). Furthermore, our results show that the width of the hip and the width of the head were overestimated to a greater extent than the width of the foot and width of the shoulders. The previous literature has suggested that estimates of emotionally salient body regions (e.g., hip and shoulder widths) could be inflated due to cognitive-affective factors (Ben-Tovim et al., 1990; Garner & Garfinkel, 1982). It would therefore be expected that women and men show more inaccuracies in estimates that are involved in the sociocultural ideal of the body of their own sex (e.g., for men, the chest and upper torso and for women, the mid and lower torso). Some of the overestimation of shoulder and hip width seen in our results might be due to body dissatisfaction. However, although some previous studies have found that women overestimated the width of the hips (Gorham & Hundleby, 1988; Pierloot & Houben, 1978), other studies reported that men and women consistently overestimated body widths of the various body regions to the same extent (Dolan, Birtchnell, & Lacey, 1987; Gardner & Bokenkamp, 1996; McCabe et al., 2006; J. K. Thompson & Thompson, 1986). The overestimation of head width is likely not related to psychosocial factors. Bianchi, Savardi, and Bertamini (2008) suggest that there might be an estimation bias toward head enlargement with normal proprioceptive information independent of whether visual information is provided or not. In Experiment 2, we asked whether this overestimation of body part widths could partly be due to generally overestimating dimensions in a metric size estimation task. Furthermore, the greater overestimation for certain body parts could also be in part due to viewing and estimating dimensions in our experimental setup, that is, due to the distance to the stimuli, the viewing angles, or the use of a mirror.

## Experiment 2

The goal of Experiment 2 was to determine whether the widths of noncorporeal objects would be similarly overestimated as body parts. In Experiment 1, participants overestimated the size of all body parts. Is part of this overestimation due to the metric size estimation method use or due to our experimental setup? To investigate this question, participants estimated the width of four objects, strips of paper, when viewed in a mirror or when viewed without a mirror, using the same visual matching task as in Experiment 1. If dimensions are generally overestimated in metric size estimation tasks or in the viewing conditions of our experimental setup, the object widths should be similarly overestimated as the widths of the body parts in Experiment 1.

### Methods

#### Participants

Thirty (17 female, 13 male) people from the Tübingen community participated in the experiment. All participants were naive to the purpose of the experiment and had normal or corrected-to-normal vision. The experiment was approved by the ethics committee of the University of Tübingen and was performed in accordance with the Declaration of Helsinki. Participants gave written informed consent and were compensated with €8 per hour for their participation.

#### Apparatus

**Figure 3.**
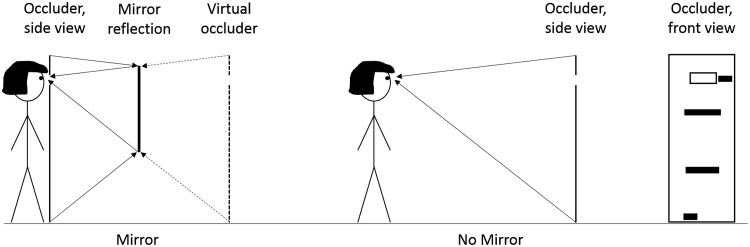
Overview of the two experimental conditions in Experiment 2. Participants either estimated the object widths attached to the occluder with visual feedback in the mirror available or by directly looking at the objects without a mirror.

The objects were four strips of paper with a length of 10 cm, 15 cm, 30 cm, and 45 cm and a width of 5 cm. The lengths of the strips of paper were roughly based on the average width of participants’ body parts (foot, hips, shoulders, and head) in Experiment 1. In the Mirror condition, participants viewed the objects in a 34 cm × 110 cm (width × height) sized mirror, mounted on a wall with a floor offset of 70 cm at a distance of 1.75 m from the viewers’ location. The objects were placed on an occluder (2 m tall, 1 m wide) at the height of each participant’s body part (foot, hips, shoulders, and head). The occluder was positioned between the participant and the mirror and contained an 18 cm × 12 cm (width × height) hole that could be adjusted in height such that it was centered around each participant’s eyes. The occluder allowed participants to view through and see the reflection of the objects attached to the other side of the occluder (Figure 3, left). In the No Mirror condition, participants stood 3.5 m from the objects that were attached to the occluder in the same way as in the Mirror condition to equate visual angle size across conditions (Figure 3, right). For the visual matching task, a standard 3 m steel tape measure was used with metric values displayed only on one side.

#### Design

A mixed design was implemented. Condition (Mirror and No Mirror) was manipulated between participants; half of the participants viewed the objects reflected through a mirror and the other half without the mirror. All participants estimated the width of each object four times, twice with the tape measure starting small and twice starting long. The order of estimates was completely randomized without restriction.

#### Procedure

After filling out the informed consent form, participants were randomly assigned to one of the two conditions. They then estimated the widths of the objects using the same visual matching task as in Experiment 1. The experiment lasted approximately 30 min.

### Analysis

Similar to the calculation of the BPI in Experiment 1, an object perception index (OPI) was calculated for getting a measure of the accuracy of the object width estimation: OPI = (estimated size/actual size) × 100. As in Experiment 1, a multilevel regression analysis was conducted. The OPI was regressed onto the objects placed at the height of the different body parts (foot, hip, shoulder, and head) and the condition (Mirror and No Mirror). All factors were allowed to interact. Specifically, the following mixed-effects model was fitted (in Wilkinson notation; Wilkinson & Rogers, 1973): OPI ∼ objects + condition + objects: condition + (1|participant). The reported results are with Satterthwaite approximation for degrees of freedom. *t* Test were used for planned comparisons; effect sizes are reported as Cohen’s *d*.

### Results

**Figure 4.**
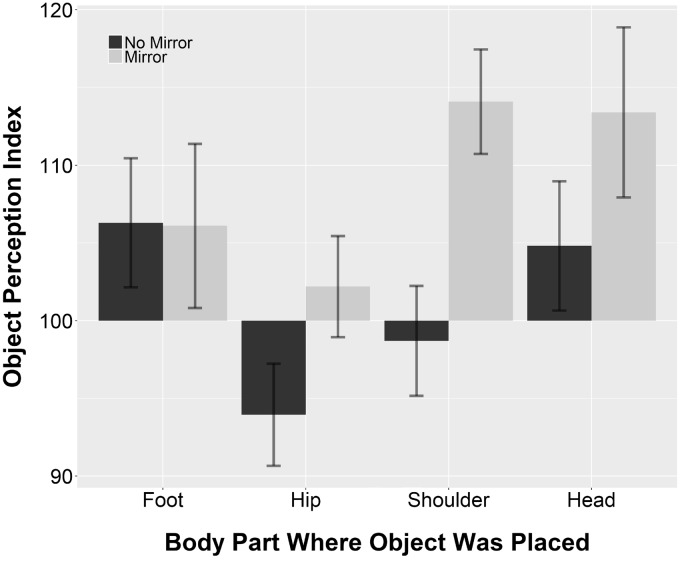
Object perception index for objects placed at the height of each participant’s right foot, hip, shoulder, and head when viewed directly (no mirror) or when viewed in a mirror (mirror) in Experiment 2. Values higher than 100 indicate overestimation in terms of percentage of the actual size. Error bars represent one standard error from the mean.

The analysis revealed a main effect of objects, *F*(3, 84) = 6.95, *p* < .001. However, planned comparisons using independent *t* tests with Bonferroni *p* value corrections showed that there was no significant difference in OPI across the objects placed at the height of the different body parts. There was also no main effect of condition, *F*(1, 28) = 2.61, *p* = .12. The accuracy of object width estimation was on average similar across the Mirror and No Mirror condition. However, the interaction between objects and condition was significant, *F*(3, 84) = 3.11, *p* = .03. The results are shown in Figure 4. Planned comparisons were run for each of the four objects to test whether the OPI differed between the Mirror and No Mirror condition. For the object placed at shoulder height, the OPI was significantly larger in the Mirror condition (*M* = 114.09, *SD* = 13.01) as compared with the No Mirror condition (*M* = 98.7, *SD* = 13.69), *t*(28) = 3.16, *p* = .003, *d* = 1.15. For all other objects, there was no significant difference in OPI between the Mirror and No Mirror conditions.

Using one-sample *t* tests, the OPI at each object height was tested to a value of 100 (accurate width estimation). The OPI of objects placed at shoulder and head height significantly differed from 100, shoulder: *t*(29) = 2.29, *p* = .03, *d* = 0.42; head: *t*(29) = 2.62, *p* = .01, *d* = 0.48. This effect, however, seems to be driven by a greater overestimation of the object width when viewing it in a mirror. The OPI of objects placed at foot and hip height was not different from 100, foot: *t*(29) = 1.88, *p* = .07, *d* = 0.34; hip: *t*(29) = −0.81, *p* = .43, *d* = 0.15.^2^

### Discussion

The results of Experiment 2 show that the width of objects placed at foot and hip height was estimated accurately, but the width of objects placed at shoulder and head height was overestimated. Importantly, this width overestimation of objects at shoulder and head height seems to be driven by a greater overestimation of these dimensions when viewing the objects in a mirror. While the results suggest that dimensions are not generally overestimated in metric size estimation methods, they do not allow to draw conclusions about what it is about viewing objects placed at the height of the shoulder and the head in the mirror in our experimental setup that leads to a size overestimation. However, the results of Experiment 2 suggest that part of the overestimation of body part widths in Experiment 1 may be due to biases when viewing or estimating the size of one’s body parts in the mirror. Notably, across the viewing conditions, the width of objects placed at the height of the foot and the hips was estimated accurately, whereas the width of the foot and hips in Experiment 1 was significantly overestimated to a degree of around 6% and 23%. The width of objects placed at the height of the shoulder and the head in Experiment 2 was overestimated by around 6% and 9%, compared to an overestimation of 12% for the shoulder width and 32% for the head width in Experiment 1.

## General Discussion

This research aimed at investigating how different visual experiences when looking at one’s own body may contribute to the conscious representation of one’s bodily dimensions. Our results of Experiment 1 demonstrate that body width estimates with visual access in the mirror available, from a first-person perspective, and with no visual access, were consistent. These findings are in line with previous research showing that visual feedback in a mirror did not improve the accuracy of estimating own bodily dimensions when compared to body size estimates from memory (Ben-Tovim & Walker, 1990; Farrell et al., 2003). This suggests that people might use a common body representation when making body size estimates under different viewing conditions and without visual access. Furthermore, our results show that body part widths were overall overestimated, and the hip and head width was overestimated to a larger degree than the foot and shoulder width. The results of Experiment 2 demonstrate that noncorporal object widths were accurately estimated for objects placed at the height of the foot and the hips but were overestimated for objects located at the height of the shoulder and the head. This overestimation of shoulder and head width, however, seems to be driven by larger estimates when viewing the objects in the mirror relative to when viewing the objects directly.

We found that participants generally overestimated the width of their body parts. This overestimation is in line with the previous literature showing that bodily dimensions tend to be overestimated in metric body size estimation tasks also in healthy participants (Mölbert et al., 2017). Mölbert et al. (2017) has argued that this overestimation of body dimensions in metric tasks might be due to the tasks assessing not only explicit knowledge about own body dimensions but also implicit body representations that rely on somatosensation. Implicit body representations, on the other hand, have been argued to be massively distorted in size and seem to be characterized by i.a. an overestimation of body part widths (Longo & Haggard, 2010).

Furthermore, our results show that hip and head width was overestimated to a greater extent than shoulder and foot width. Using a similar visual matching task than the one used in our study, Bianchi et al. (2008) also found a significant overestimation of head size both when estimating head height without visual access and with visual feedback in a mirror available, in their case, overestimation was, however, reduced with visual feedback. In line with the claim that metric methods might also recruit distorted implicit body representations, Bianchi et al. suggest that there might be a bias toward enlargement of heads with normal proprioceptive information that is independent of whether visual information is available or not. Interestingly, Bianchi et al. additionally analyzed paintings of portraits of self and others and found that painters drew their own head larger than other people’s heads.

Several researchers have argued that performance in body size estimation tasks can be influenced by cognitive-affective factors (Ben-Tovim & Walker, 1990; Bowden et al., 1989; Garner & Garfinkel, 1982). Thus, estimates of emotionally salient body region, such as the hips and shoulders, might be inflated due to body dissatisfaction, and this could also contribute to the overestimation of body part widths seen in Experiment 1. As our experiment was not designed for looking at sex differences in the accuracy of body width estimates, but at the role of visual information, it remains up to future research to determine if and how psychosocial factors correlate with estimates of own body size. An influence of attitudinal factors on the accuracy of body size estimates should theoretically reveal sex differences, with more overestimation of the hip region for women and more overestimation of the shoulder region for men according to societal beauty ideals. However, several studies found no sex difference in body width estimates of the different body regions (Dolan et al., 1987; Gardner & Bokenkamp, 1996; McCabe et al., 2006; J. K. Thompson & Thompson, 1986).

Perception of own body size can also be influenced by the bodies that one has been exposed to in the past. There is consistent evidence that familiarity or expertise with different body sizes modulates how bodies are perceived. For example, aftereffects result from prolonged or repeated exposure to a given feature and manifest themselves in a subsequent shift of perception of this feature into the direction opposite of the adapted feature (P. Thompson & Burr, 2009). Such biases in perception of bodies have been found in adaptation experiments in terms of body normality (Glauert, Rhodes, Byrne, Fink, & Grammer, 2009), body identity (Rhodes, Jeffery, Boeing, & Calder, 2013), and attractiveness (Winkler & Rhodes, 2005). Similarly, Hummel et al. (2013) found that after adaptation to a thin body, participants judged a picture depicting a thinner version of themselves as most realistic and after adapting to a fat body, a fatter version as most realistic. Aftereffects were also found to transfer across self and other identity, suggesting that they might also explain the sociocultural influence on disturbances of body image as repeated media exposure to extreme thin bodies should make bodies appear fatter (Brooks, Mond, Stevenson, & Stephen, 2016). Repeated visual exposure to distorted mirrors, an extreme case would be funhouse mirrors, could therefore also affect how one’s own body size is perceived and estimated.

In Experiment 2, we tested whether the width overestimation was specific to body parts or might be partly due to the metric method used or aspects of our experimental setup and would therefore also affect estimates of noncorporeal objects. The results demonstrate that the width of objects placed at the height of the foot and the hip was accurately estimated, suggesting that dimensions are not generally overestimated in a metric size estimation task, whereas there is a large body of literature on estimates of object sizes within mirrors, this study provides a further contribution to this previous literature by manipulating object height and size to match a situation of judging body part widths. We only found some overestimation of object widths when looking in the mirror, specifically at the height of the shoulder and the head. However, the magnitude of this overestimation was much less than for the body size estimates in Experiment 1.

Previous work has asked participants to estimate the size of objects, as they appear on the mirror surface (Lawson & Bertamini, 2006) or when viewed through a window (Lawson, Bertamini, & Liu, 2007). Those results show that participants have difficulties estimating the object size as it appears on the mirror surface (estimates should be one half of the actual width) and tend to make estimates close to the true size of the object. Furthermore, size estimates on the mirror surface were found to depend partially on the distance between the viewpoint (participants’ eyes) and the horizontal displacement of the object. For both mirrors and windows, the physical size of an object was, however, accurately estimated. Our results demonstrate that the width of objects placed at the height of the shoulder and the head was overestimated when participants were asked to estimate their actual width, when seeing the object reflection in a mirror as compared with when directly viewing the objects. The difference in results could be due to having a size reference, namely, the mirror reflection of one’s own body in the study by Lawson et al. (2007), whereas participants in our study had to judge the size of an object without any familiar size they could use as a reference. However, lack of familiar size cues cannot explain why the width of objects placed at the height of the foot and the hip was accurately estimated. There might be a relationship between vertical displacement between the object being judged and the viewpoint. Although this mirror effect cannot entirely explain the overestimation of body part widths of Experiment 1, it suggests that visual experience of one’s body in the mirror and potential distortions thereof could also be reflected in the mental image of own body dimensions.

Another prominent visual experience of one’s own body is viewing it in a picture. Docteur et al. (2012) found that participants accurately estimated their body size when they had to adjust an image so it matched a printed photograph of themselves. Although perception of body size in photographs provides another interesting field for understanding body image distortions, the size of one’s body in an image also relies heavily on familiar size cues and indirect extrapolation of one’s size given the environment. Moreover, in most such depictive body size estimation tasks where the reference is an entire body that has to be matched to one’s own body shape, the body stimulus can only be adjusted as a whole, most often in body weight, and not independently for single body parts. Thus, estimates are probably influenced by relative proportions of the body and the overall body shape.

Another possible indirect experience of one’s body size is how we interact with the world around us by stepping up onto objects or passing in between obstacles. Previous work has shown that much of our interpretation of the spatial layout is scaled to the size of one’s body (Warren & Whang, 1987). It would be interesting to see whether and how manipulating one’s ability to act in the world alters subsequent estimations of one’s body width in body size estimation tasks.

Finally, this study has some limitations. First, we only examined the visual estimation of the width of four body parts, and second, we did not use a measure for body dissatisfaction, especially with regard to the body parts that were estimated. Future research should include more measures of the attitudinal component of body image to understand the contribution of visual and cognitive factors to body size estimation. Another limitation of our study is that the overestimation of object widths in the mirror of Experiment 2 cannot be traced to the size or the height of the objects because the object widths were matched to the body part widths of Experiment 1 and the objects were placed at the height of the respective body part for each participant. Future studies should test size estimation of the same object at different heights. Furthermore, similar methods could be used to understand how other experiences with different senses influence body size estimation, for example, how mirror exposure and simultaneous tactile stimulation alter visual size estimates.

## Notes

1 We ran a second multilevel analysis with participant sex included. The analysis revealed a main effect of body part and an interaction of gender and body part, *F*(3, 117.07) = 3.3, *p* = .02. Planned comparisons showed no sex difference in estimates for foot width, hip width, and shoulder width (all *p* > .26), but for head width, *t*(22.1) = 2.16, *p* = .04, *d* = 0.82 (BPI females: *M* = 1.39, *SD* = 0.23; BPI males: *M* = 1.2, *SD* = 0.25)As our experiment was not designed for investigating sex differences and there was an unequal number of males and females in the three different experimental conditions, we, however, do not want to make strong claims about this finding.

2 As in Experiment 1, a second mixed-effects model was run with participant sex included. There was no significant effect of participant sex, *F*(1, 26) = 0.19, *p* = .67, and none of the interactions were significant (all *p* values > .16).

## References

[bibr1-2041669518796853] Bates, D., Mächler, M., Bolker, B., & Walker, S. (2014). Fitting linear mixed-effects models using lme4. *arXiv preprint*, arXiv:1406.5823.

[bibr2-2041669518796853] Ben-Tovim D. I. Walker M. K. (1990) Effect of a mirror on body-size estimation. Perceptual and Motor Skills 71: 1151–1154. 2087369 10.2466/pms.1990.71.3f.1151

[bibr3-2041669518796853] Ben-Tovim D. I. Walker M. K. Murray H. Chin G. (1990) Body size estimates: Body image or body attitude measures? International Journal of Eating Disorders 9: 57–67.

[bibr4-2041669518796853] Bianchi I. Savardi U. Bertamini M. (2008) Estimation and representation of head size (people overestimate the size of their head—Evidence starting from the 15th century). British Journal of Psychology 99: 513–531. 18471345 10.1348/000712608X304469

[bibr5-2041669518796853] Bowden P. Touyz S. Rodriguez P. Hensley R. Beumont P. (1989) Distorting patient or distorting instrument? Body shape disturbance in patients with anorexia nervosa and bulimia. The British Journal of Psychiatry 155: 196–201. doi: 10.1192/bjp.155.2.196. 2597915 10.1192/bjp.155.2.196

[bibr6-2041669518796853] Brooks K. R. Mond J. M. Stevenson R. J. Stephen I. D. (2016) Body image distortion and exposure to extreme body types: Contingent adaptation and cross adaptation for self and other. Frontiers in Neuroscience 10: 334. 27471447 10.3389/fnins.2016.00334 PMC4946181

[bibr7-2041669518796853] Cappon D. Banks R. (1968) Distorted body perception in obesity. The Journal of Nervous and Mental Disease 146: 465–467. 5677023 10.1097/00005053-196806000-00005

[bibr8-2041669518796853] Delinsky S. S. Wilson G. T. (2006) Mirror exposure for the treatment of body image disturbance. International Journal of Eating Disorders 39: 108–116. doi: 10.1002/eat.20207. 16231342 10.1002/eat.20207

[bibr9-2041669518796853] Dijkerman H. C. De Haan E. H. (2007) Somatosensory processing subserving perception and action: Dissociations, interactions, and integration. Behavioral and Brain Sciences 30: 224–230. doi: 10.1017/S0140525X07001641. 10.1017/S0140525X07001392 17705910

[bibr10-2041669518796853] Docteur A. Urdapilleta I. Duarte L. R. (2012) The role of cognitive factors in body-size perception and recall-size estimation in normal-weight women. Revue Européenne de Psychologie Appliquée/European Review of Applied Psychology 62: 129–135.

[bibr11-2041669518796853] Dolan B. M. Birtchnell S. A. Lacey J. H. (1987) Body image distortion in non-eating disordered women and men. Journal of Psychosomatic Research 31: 513–520. 3668888 10.1016/0022-3999(87)90009-2

[bibr12-2041669518796853] Farrell C. Shafran R. Fairburn C. G. (2003) Body size estimation: Testing a new mirror-based assessment method. International Journal of Eating Disorders 34: 162–171. doi: 10.1002/eat.10174. 12772182 10.1002/eat.10174

[bibr13-2041669518796853] Gardner R. M. Bokenkamp E. D. (1996) The role of sensory and nonsensory factors in body size estimations of eating disorder subjects. Journal of Clinical Psychology 52: 3–15. 8682909 10.1002/(SICI)1097-4679(199601)52:1<3::AID-JCLP1>3.0.CO;2-X

[bibr14-2041669518796853] Gardner R. M. Brown D. L. (2014) Body size estimation in anorexia nervosa: A brief review of findings from 2003 through 2013. Psychiatry Research 219: 407–410. 25023364 10.1016/j.psychres.2014.06.029

[bibr15-2041669518796853] Gardner R. M. Gallegos V. Martinez R. Espinoza T. (1989) Mirror feedback and judgments of body size. Journal of Psychosomatic Research 33: 603–607. 2795532 10.1016/0022-3999(89)90067-6

[bibr16-2041669518796853] Gardner R. M. Martinez R. Espinoza T. Gallegos V. (1988) Distortion of body image in the obese: A sensory phenomenon. Psychological Medicine 18: 633–641. doi: 10.1017/S003329170000831X. 3186867 10.1017/s003329170000831x

[bibr17-2041669518796853] Garfinkel P. E. Moldofsky H. Garner D. M. Stancer H. C. Coscina D. V. (1978) Body awareness in anorexia nervosa: Disturbances in body image and satiety. Psychosomatic Medicine 40: 487–498. 734025 10.1097/00006842-197810000-00004

[bibr18-2041669518796853] Garner D. M. Garfinkel P. E. (1982) Body image in anorexia nervosa: Measurement, theory and clinical implications. The International Journal of Psychiatry in Medicine 11: 263–284. 10.2190/r55q-2u6t-lam7-rqr7 7309395

[bibr19-2041669518796853] Glauert R. Rhodes G. Byrne S. Fink B. Grammer K. (2009) Body dissatisfaction and the effects of perceptual exposure on body norms and ideals. International Journal of Eating Disorders 42: 443–452. doi: 10.1002/eat.20640. 19115365 10.1002/eat.20640

[bibr20-2041669518796853] Gorham S. J. Hundleby J. D. (1988) Present body perception and prior weight reduction in young adult women. International Journal of Eating Disorders 7: 407–411.

[bibr21-2041669518796853] Grabe S. Ward L. M. Hyde J. S. (2008) The role of the media in body image concerns among women: A meta-analysis of experimental and correlational studies. Psychological Bulletin 134: 460–476. 18444705 10.1037/0033-2909.134.3.460

[bibr22-2041669518796853] Home R. L. Van Vactor J. C. Emerson S. (1991) Disturbed body image in patients with eating disorders. The American Journal of Psychiatry 148: 211–215. 1987820 10.1176/ajp.148.2.211

[bibr23-2041669518796853] Hummel D. Rudolf A. K. Brandi M.-L. Untch K.-H. Grabhorn R. Hampel H. Mohr H. M. (2013) Neural adaptation to thin and fat bodies in the fusiform body area and middle occipital gyrus: An fMRI adaptation study. Human Brain Mapping 34: 3233–3246. doi: 10.1002/hbm.22135. 22807338 10.1002/hbm.22135 PMC6870049

[bibr24-2041669518796853] Lawson R. Bertamini M. (2006) Errors in judging information about reflections in mirrors. Perception 35: 1265–1288. 17120845 10.1068/p5498

[bibr25-2041669518796853] Lawson R. Bertamini M. Liu D. (2007) Overestimation of the projected size of objects on the surface of mirrors and windows. Journal of Experimental Psychology: Human Perception and Performance 33: 1027–1044. 17924805 10.1037/0096-1523.33.5.1027

[bibr26-2041669518796853] Longo M. R. Haggard P. (2010) An implicit body representation underlying human position sense. Proceedings of the National Academy of Sciences 107: 11727–11732. 10.1073/pnas.1003483107 PMC2900654 20547858

[bibr27-2041669518796853] Longo M. R. Haggard P. (2012) Implicit body representations and the conscious body image. Acta Psychologica 141: 164–168. doi: 10.1016/j.actpsy.2012.07.015. 22964057 10.1016/j.actpsy.2012.07.015

[bibr28-2041669518796853] McCabe M. P. Ricciardelli L. A. Sitaram G. Mikhail K. (2006) Accuracy of body size estimation: Role of biopsychosocial variables. Body Image 3: 163–171. 18089219 10.1016/j.bodyim.2006.01.004

[bibr29-2041669518796853] Mölbert S. C. Klein L. Thaler A. Mohler B. J. Brozzo C. Martus P. Giel K. E. (2017) Depictive and metric body size estimation in anorexia nervosa and bulimia nervosa: A systematic review and meta-analysis. Clinical Psychology Review 57: 21–31. doi: 10.1016/j.cpr.2017.08.005. 28818670 10.1016/j.cpr.2017.08.005

[bibr30-2041669518796853] Mölbert S. C. Thaler A. Mohler B. J. Streuber S. Romero J. Black M. J. Giel K. E. (2018) Assessing body image in anorexia nervosa using biometric self-avatars in virtual reality: Attitudinal components rather than visual body size estimation are distorted. Psychological Medicine 48: 642–653. doi: 10.1017/S0033291717002008. 28745268 10.1017/S0033291717002008 PMC5964466

[bibr31-2041669518796853] Myers T. A. Crowther J. H. (2009) Social comparison as a predictor of body dissatisfaction: A meta-analytic review. Journal of Abnormal Psychology 118: 683–698. 19899839 10.1037/a0016763

[bibr32-2041669518796853] Norris D. L. (1984) The effects of mirror confrontation on self-estimation of body dimensions in anorexia nervosa, bulimia and two control groups. Psychological Medicine 14: 835–842. 6599508 10.1017/s0033291700019802

[bibr33-2041669518796853] Øverås M. Kapstad H. Brunborg C. Landrø N. I. Lask B. (2014) Memory versus perception of body size in patients with anorexia nervosa and healthy controls. European Eating Disorders Review 22: 109–115. doi: 10.1002/erv.2276. 24590562 10.1002/erv.2276

[bibr34-2041669518796853] Paillard, J. (1999). Body schema and body image—A double dissociation. In G. N. Gantchev, S. Mori, & J. Massion (Eds.) *Motor Control, Today and Tomorrow*, 197–214. Sofia, Bulgaria: Academic Publishing House.

[bibr35-2041669518796853] Pierloot R. Houben M. (1978) Estimation of body dimensions in anorexia nervosa. Psychological Medicine 8: 317–324. doi: 10.1017/S0033291700014367. 652902 10.1017/s0033291700014367

[bibr36-2041669518796853] Piryankova I. V. Stefanucci J. K. Romero J. De La Rosa S. Black M. J. Mohler B. J. (2014) Can I recognize my body’s weight? The influence of shape and texture on the perception of self. ACM Transactions on Applied Perception 11: 13 . doi: 10.1145/2641568.

[bibr37-2041669518796853] Rhodes G. Jeffery L. Boeing A. Calder A. J. (2013) Visual coding of human bodies: Perceptual aftereffects reveal norm-based, opponent coding of body identity. Journal of Experimental Psychology: Human Perception and Performance 39: 313–317. 23398261 10.1037/a0031568

[bibr38-2041669518796853] Shafran R. Fairburn C. G. (2002) A new ecologically valid method to assess body size estimation and body size dissatisfaction. International Journal of Eating Disorders 32: 458–465. doi: 10.1002/eat.10097. 12386910 10.1002/eat.10097

[bibr39-2041669518796853] Slade P. D. (1994) What is body image? Behaviour Research and Therapy 32: 497–502. doi: 10.1016/0005-7967(94)90136-8. 8042960 10.1016/0005-7967(94)90136-8

[bibr40-2041669518796853] Slade P. D. Russell G. (1973) Awareness of body dimensions in anorexia nervosa: Cross-sectional and longitudinal studies. Psychological Medicine 3: 188–199. doi: 10.1017/S0033291700048510. 4715850 10.1017/s0033291700048510

[bibr41-2041669518796853] Smeets M. A. (1997) The rise and fall of body size estimation research in anorexia nervosa: A review and reconceptualization. European Eating Disorders Review 5: 75–95. doi: 10.1002/(SICI)1099-0968(199706)5:2<75::AID-ERV190>3.0.CO;2-A.

[bibr42-2041669518796853] Thaler A. Geuss M. N. Mölbert S. C. Giel K. E. Streuber S. Romero J. Mohler B. J. (2018) Body size estimation of self and others in females varying in BMI. PLoS One 13: e0192152 . doi: 10.1371/journal.pone.0192152. 29425218 10.1371/journal.pone.0192152 PMC5806871

[bibr43-2041669518796853] Thompson J. K. Coovert M. D. Stormer S. M. (1999) Body image, social comparison, and eating disturbance: A covariance structure modeling investigation. International Journal of Eating Disorders 26: 43–51. 10349583 10.1002/(sici)1098-108x(199907)26:1<43::aid-eat6>3.0.co;2-r

[bibr44-2041669518796853] Thompson J. K. Thompson C. M. (1986) Body size distortion and self-esteem in asymptomatic, normal weight males and females. International Journal of Eating Disorders 5: 1061–1068.

[bibr45-2041669518796853] Thompson P. Burr D. (2009) Visual aftereffects. Current Biology 19: R11–R14. 19138580 10.1016/j.cub.2008.10.014

[bibr46-2041669518796853] Tovée M. J. Benson P. J. Emery J. L. Mason S. M. Cohen-Tovée E. M. (2003) Measurement of body size and shape perception in eating-disordered and control observers using body-shape software. British Journal of Psychology 94: 501–516. doi: 10.1348/000712603322503060. 14687458 10.1348/000712603322503060

[bibr47-2041669518796853] Warren W. H. Whang S. (1987) Visual guidance of walking through apertures: Body-scaled information for affordances. Journal of Experimental Psychology: Human Perception and Performance 13: 371–383. 2958586 10.1037//0096-1523.13.3.371

[bibr48-2041669518796853] Wilkinson G. Rogers C. (1973) Symbolic description of factorial models for analysis of variance. Journal of the Royal Statistical Society, Series C (Applied Statistics) 22: 392–399.

[bibr49-2041669518796853] Winkler C. Rhodes G. (2005) Perceptual adaptation affects attractiveness of female bodies. British Journal of Psychology 96: 141–154. doi: 10.1348/000712605X36343. 15969827 10.1348/000712605X36343

